# Cryptotephras: the revolution in correlation and precision dating^1^

**DOI:** 10.1002/jqs.2766

**Published:** 2015-03-30

**Authors:** SIWAN M DAVIES

**Affiliations:** Department of Geography, College of Science, Swansea UniversitySwansea, Wales, UK

**Keywords:** correlation, cryptotephra deposits, geochemical signatures, regional frameworks, tephrochronology

## Abstract

From its Icelandic origins in the study of visible tephra horizons, tephrochronology took a remarkable step in the late 1980 s with the discovery of a ca. 4300-year-old microscopic ash layer in a Scottish peat bog. Since then, the search for these cryptotephra deposits in distal areas has gone from strength to strength. Indeed, a recent discovery demonstrates how a few fine-grained glass shards from an Alaskan eruption have been dispersed more than 7000 km to northern Europe. Instantaneous deposition of geochemically distinct volcanic ash over such large geographical areas gives rise to a powerful correlation tool with considerable potential for addressing a range of scientific questions. A prerequisite of this work is the establishment of regional tephrochronological frameworks that include well-constrained age estimates and robust geochemical signatures for each deposit. With distal sites revealing a complex record of previously unknown volcanic events, frameworks are regularly revised, and it has become apparent that some closely timed eruptions have similar geochemical signatures. The search for unique and robust geochemical fingerprints thus hinges on rigorous analysis by electron microprobe and laser ablation-inductively coupled plasma-mass spectrometry. Historical developments and significant breakthroughs are presented to chart the revolution in correlation and precision dating over the last 50 years using tephrochronology and cryptotephrochronology.

## Introduction

### Historical developments and landmark discoveries

Little did we realize that tephrochronology and, in particular, the search for cryptotephra deposits, would become such an invaluable technique for Quaternary studies. This technique has long been prominent in volcanic areas, such as Iceland, New Zealand and Japan, but little did we know of its potential and promise for more distal regions. Here, I chart the development and advances that have brought cryptotephrochronology to the forefront of Quaternary science in Europe. The Quaternary Research Association (https://www.qra.org.uk) and its members have made significant contributions to this work and thus it is fitting that we celebrate the achievements in the volume to mark 50 years of the Association. After the Eyjafjöll eruption in 2010 we are all fully aware how a little goes a long way in terms of ash dispersal, but the same also applies in relation to the revolutionary advances and far-reaching scientific applications of cryptotephrochronology. I refer readers to the reviews of Lowe (2011, in revision) for further information on the all-encompassing tephrochronology toolkit. This publication takes its sole inspiration from the developments in cryptotephra studies with a focus on Europe.

Although descriptions of tephra deposits within soil sections in Iceland can be traced back to the 17th century, it was not until the 1930 s that these deposits were actually investigated in detail (Thórarinsson, 1981b,1981c). While attempting to apply the pollen analytical method in Iceland, Sigurđur Thórarinsson, a Stockholm University doctoral student, working under the guidance of Lennart von Post (the founder of palynology), realized the potential of tracing and correlating common tephra deposits over great distances. As such, the value of tephra horizons became apparent to Thórarinsson, not only for providing dated horizons for his pollen work but also for constraining occupation and abandonment of human settlements in Iceland. In his doctoral thesis, he proposed the term tephra (from the Greek word τέϕρα used by Aristotle) and tephrochronology ‘as an international term to designate a geological chronology based on the measuring, interconnecting, and dating of volcanic ash layers in soil profiles’ (Thórarinsson, 1944; p. 204). Early applications of this technique are also documented from other parts of the globe e.g. Tierra del Fuego (Auer, 1965; following fieldwork in the 1920 s), Chile (Larsson, 1936), the Mediterranean region (Mellis, 1954), New Zealand (Grange, 1931) and Japan (Uragami *et al*., 1933). The start of our fascination with far-travelled tephra deposits, however, lies exclusively with Icelandic ashes and their dispersal to the European continent. Indeed, the first to map the extent of a tephra deposit beyond Iceland’s shores was Mohn (1878), a meteorologist who compiled people’s observations of Askja 1875 fall-out in Norway and Sweden (Thórarinsson, 1981b). Over 100 years passed, however, before the first discovery was made of an Icelandic tephra deposit on the British mainland (Dugmore, 1989).

Following Thórarinsson’s pioneering work, the prominence and fascination with distal deposits in Europe developed in two stages. In the 1950 s and 1960 s, the first stage centred on Scandinavian studies. Noe-Nygaard (1951) reported the discovery of sub-fossil Hekla pumice in Denmark (most likely Hekla 4) and Persson (1966, 1967, 1968, 1971), who was inspired by Thórarinsson, identified several deposits, invisible to the naked eye, within Swedish, Norwegian and Faeroes peat bogs. Although his attributions lacked geochemical verification, Persson identified Hekla 3, Hekla 4, Askja 1875 and Öraefajökull 1362 and in his 1971 publication, he wrote ‘It is also probable that the ash units identified in this study occur over wider areas, even outside Scandinavia. It would be of interest, for example, to investigate mires on the Shetland Islands and in Scotland in this connection. Eventual application to the stratigraphy of the North Atlantic sediments may be anticipated’ (p. 30). Persson’s predictions were, however, slow to progress as work in the 1970 s and 1980 s focused largely on visible tephra horizons in the North Atlantic marine realm and coastal areas of Norway (e.g. Ruddiman and Glover, 1972; Sigurdsson, 1982; Mangerud *et al.*, 1986, 1984; Kvamme *et al.*, 1989).

In the late 1980 s, a volcanic ash layer identified in a Younger Dryas deposit on the continental shelf in the North Sea (Long and Morton, 1987) echoed Persson’s predictions and within a few years Andrew Dugmore published the first discovery of an Icelandic tephra deposit in Scotland (Dugmore, 1989). Dugmore’s discovery of a tephra deposit that was invisible to the naked eye from the Hekla 4 eruption, within a peat sequence from Caithness, Scotland, was a significant breakthrough. In 2014, 25 years later, Dugmore’s seminal discovery and subsequent tephra-based research were recognized by the international tephra community by the award of honorary life membership of the International focus group on tephrochronology and volcanism (INTAV).

By now, these deposits have become known as cryptotephras (Lowe and Hunt, 2001), but they have also been referred to as micro-tephra (Lowe and Turney, 1997). These deposits contain a low concentration of volcanic glass shards and as a result leave no macroscopic imprint of their presence in a core sequence or exposure. Consequently, these hidden deposits can only be detected by the systematic application of extraction techniques performed in the laboratory, and the paper by Dugmore (1989) marks the start of the second phase that led to the rapid expansion and application of tephrochronology in distal areas. The foundations for the future were set in this landmark paper: ‘The priority would seem to be the search of U.K. peats and lake sediments for traces of the 12 other Holocene eruptions known to have spread ashes far beyond Iceland’s shores, because the more detailed the tephra stratigraphy in the U.K., the more useful it will be’ (Dugmore, 1989; p. 171). Over the next few years, a flurry of publications reported the discovery of Icelandic cryptotephra deposits in Scotland (Dugmore and Newton, 1992; Dugmore *et al.*, 1995, 1996), the Faroe Islands (Dugmore and Newton, 1997), Northern Ireland (Pilcher and Hall, 1992; Hall *et al.*, 1993; Pilcher *et al.*, 1995, 1996) and northern England (Pilcher and Hall, 1996). What distinguished this body of work from Persson’s was the acquisition of geochemical data that provided a fingerprint for the successful correlation of deposits. Geochemical analysis of single shards illustrated the methodological way forward.

**Figure 1:**
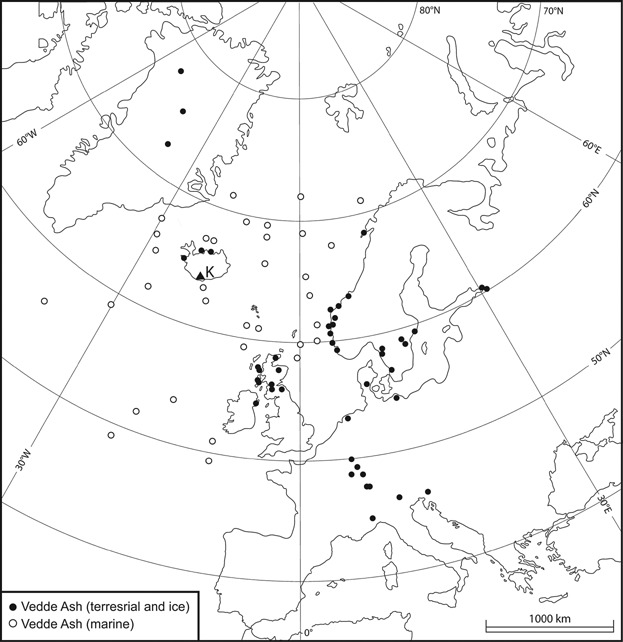
Location of palaeorecords within which the Vedde Ash has been identified. Filled circles mark the location of terrestrial and ice-core sequences and open circles represent marine occurrences. Deposition of Vedde Ash is thought to have been by iceberg rafting between 49 and 55°N and primary fallout north of 55 °N. References for the Vedde Ash occurrences are as follows: Greenland (Grönvold *et al.*, 1995; Mortensen *et al.*, 2005; Rasmussen *et al.*, 2013); Iceland (Björck *et al.*, 1992; Norddahl and Haflidason, 1992; Ingólfsson *et al.*, 1997); Norway (Mangerud *et al.*, 1984; Birks *et al.*, 1996; Bondevik *et al.*, 2001; Vorren *et al.*, 2007; Lind *et al.*, 2013); Sweden (Wastegård *et al.*, 1998; Björck and Wastegård, 1999; Wastegård *et al.*, 2000a; Schoning, 2002; Macleod *et al.*, 2014); Russian Federation (Wastegård *et al.*, 2000b); Scotland (Lowe and Turney, 1997; Turney *et al.*, 1997; Davies *et al.*, 2001; Mackie *et al.*, 2002; Ranner *et al.*, 2005; Pyne-O’Donnell, 2007; Matthews *et al.*, 2011); Northern Ireland (Turney *et al.*, 2006); Netherlands (Davies *et al.*, 2005); Denmark (Larsen and Noe-Nygaard, 2014); France, Germany and Switzerland (Blockley *et al.*, 2007; Walter-Simonnet *et al.*, 2008; Lane *et al.*, 2012a,2012c); Italy (Lane *et al.*, 2012a); Slovenia (Lane *et al.*, 2011a); North Atlantic Ocean (Ruddiman and Glover, 1972; Sigurdsson, 1982; Long and Morton, 1987; Kvamme *et al.*, 1989; Sejrup *et al.*, 1989; Sjøholm *et al.*, 1991; Koç and Jansen, 1992; Bard *et al.*, 1994; Austin *et al.*, 1995; Hunt *et al.*, 1995; Lacasse *et al.*, 1995; Lackschewitz and Wallrabe-Adams, 1997; Thornalley *et al.*, 2011).

By the latter half of the 1990 s a further development saw Chris Turney and John Lowe modify a pollen extraction technique based on density differences to isolate microscopic Vedde Ash glass shards from mineral-rich Younger Dryas sediment (Lowe and Turney, 1997; Turney, 1998). Isolating cryptotephras from peat deposits was relatively straightforward and simply required digestion of the organic-rich host material either via ashing or acid treatment (Dugmore *et al.*, 1992). Investigating the cryptotephra content within minerogenic lacustrine sediment, however, was a different matter altogether and the development of an extraction technique that exploited the relative density of a tephra deposit then precipitated somewhat of a Lateglacial tephra rush (Turney *et al.*, 1997). These advancements were not limited to the UK (see, for example, Lowe *et al.*, 2008) and within a short time Icelandic cryptotephra finds were uncovered in new areas where tephra research had always been considered futile. A notable example of this is the Vedde Ash distribution, which can now can be mapped as far east as western Russia and as far south as Switzerland, Slovenia and northern Italy (Fig. 1) (Wastegård *et al.*, 2000b; Blockley *et al.*, 2007; Lane *et al.*, 2011a). These discoveries unlocked the potential for further cryptotephra work in these regions and elsewhere, and the quest for records that contained cryptotephras from more than one volcanic area was instigated (i.e. to widen the geographical area over which correlations were possible by overlapping ash plumes from, for example, Iceland, the Eifel and the Mediterranean region in e.g. Lane *et al.*, 2011a).

In the early days of distal tephra research, key individuals at the University of Edinburgh, Queen’s University Belfast and Royal Holloway led the way and provided training and inspiration for a new generation of researchers. Indeed, the 2012 Quaternary Research Association field excursion on Holocene tephra deposits in southern and central Iceland is acknowledgment in itself of how this technique has become central to several research laboratories in the UK and NW Europe (Catt and Candy, 2014). Likewise, one particular initiative that has facilitated and supported tephrochronological developments within Europe is INTIMATE.^1^ The early focus of this group was the rapid climatic shifts of the Lateglacial period and how disparate palaeoclimatic records from the cryospheric, terrestrial and marine realm could be integrated and synchronized independently. In light of the methodological developments for isolating and pinpointing cryptotephra deposits in lacustrine sediment and peats and the identification of well-resolved Vedde Ash deposits in the ice and marine realms, the application of tephrochronology became a key recommendation of the INTIMATE group (e.g. Lowe *et al.*, 2001). By now, the INTIMATE time interval under focus has been extended back to 60 000 years and tephrochronology remains a key correlation and dating technique (e.g. Lowe *et al.*, 2008; Blockley *et al.*, 2012; Davies *et al.*, 2014, 2012). INTIMATE has also offered crucial support and encouragement to early-career researchers giving access to collaborative networks, summer training schools and an international platform for dissemination of results. It is probably fair to say that several major research projects and fellowships have been brought to fruition due to INTIMATE inspiration (e.g. RESET^2^, TRACE^3^, SMART^4^). The equivalent Australasian INTIMATE project (and specifically the NZ-INTIMATE subproject) has also seen tephrochronology become central to the development of a robust climate event stratigraphy (Barrell *et al.*, 2013; Lowe *et al.*, 2013).

**Figure 2:**
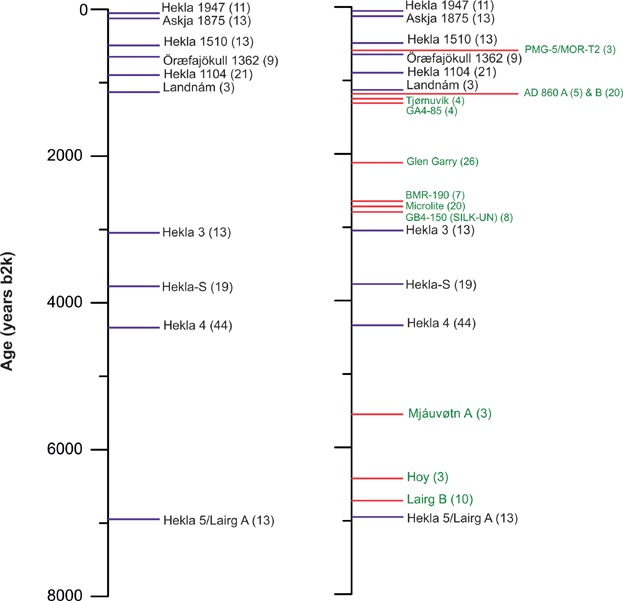
Icelandic tephrostratigraphy for the last 8000 years based on the synthesis of Lawson *et al.* (2012). Blue lines represent volcanic events and tephras that are well known and well constrained within the proximal stratigraphy in Iceland. Red lines represent new, previously unknown tephra or cryptotephra deposits identified outside of Iceland in distal settings. Numbers in parentheses represent the number of distal sites at which these tephras/cryptotephras have been found. This figure is available in colour online at wileyonlinelibrary.com.

Cryptotephra studies have, by now, been applied to a wide-range of different time intervals (e.g. Wastegård *et al.*, 2005; Brough *et al.*, 2010; Davies *et al.*, 2014; Hibbert *et al.*, 2014) and the geographical area over which Icelandic tephras have been traced is staggering. The well-known Hekla 4 and Hekla 1104 tephras have been discovered within 40 and 20 different peat deposits in NW Europe, respectively (Fig. 2) (Lawson *et al.*, 2012). The predictions outlined by Persson in the 1960 s, and Dugmore in 1989, may indeed reflect elements of tephromancy and the ability to foresee the future from the ashes!

Extending the distribution of tephra isochrons, however, is not exclusive to those of Icelandic origin. An astounding discovery by Pyne-O’Donnell *et al.* (2012) revealed how tephra from Alaskan sources have been transported 7000 km to Newfoundland. This discovery is certain to mark the start of a cryptotephra rush on the North American continent akin to the last few decades in NW Europe. What is more, one particular Alaskan tephra, the White River Ash (AD 833–850), has also been correlated to the well-known AD 860B isochron identified in Ireland (Hall and Pilcher, 2002; Jensen *et al.*, 2014) and beyond. Further afield, Cullen *et al.* (2014) demonstrate how Italian, Hellenic and Turkish tephras are preserved in a Black Sea core, opening up the possibilities for tracing tephras from these sources further east. Moreover, the discovery of fallout material from Pacific arc volcanoes in Greenland ice presents an exciting opportunity for Atlantic–Pacific correlations (Bourne *et al.*, 2014). Several of these new tephra discoveries originate from, what are believed to be, moderate-sized eruptions in terms of eruptive volume (e.g. Jensen *et al.*, 2014). Eruptions of this magnitude have been overlooked in the past in terms of both their reach for correlation purposes and their impact in terms of ash-related hazard assessments (Davies *et al.*, 2010a; Jensen *et al.*, 2014). Such ultra-distal discoveries raise the cryptotephra work to a new level with hemispheric correlations beyond the European continent now a real possibility.

## Methodological developments: extracting, fingerprinting and correlating cryptotephra deposits

### Cryptotephra extraction techniques

Cryptotephra extraction techniques are relatively simple to execute but somewhat labour-intensive. The most commonly applied techniques largely involve removing the vast majority of the host material to concentrate the fine-grained glass shards. Methodologies are specifically tailored so that a vast range of depositional environments can be explored. Further details on each of these techniques are available in the indicative references provided in Table1. The ashing and density separation techniques are probably the most commonly used techniques and can be executed with standard laboratory equipment (e.g. furnace, centrifuge, sieving equipment and slide preparation facilities). Each analyst will typically use a combination of the available techniques and modify these according to the nature of the sediment under investigation. The most common approach is to first explore low-resolution contiguous samples (e.g. 5- or 10-cm contiguous samples) and then undertake a targeted higher-resolution search if glass shards are detected. This is the most time-effective approach to investigate an entire sequence. Nevertheless, cryptotephra analysts will be more than familiar with the significant time investment required to search for these ‘needles in haystacks’. Some samples and, at times, entire sequences will reveal no trace of glass shards after days or weeks in the laboratory. As a result any positive discovery heralds a mini-celebration! It is fair to say that cryptotephra analysts typically develop and share some common traits, with most being methodical and patient and, above all, showing strong determination and perseverance.

**Table 1:** Destructive and non-destructive techniques developed for extracting and isolating cryptotephra deposits. Some indicative references are included. For a comprehensive review of extraction techniques see Gehrels *et al.* (2008) and Lowe (2011)

Extraction technique	Host material	Indicative references
Destructive techniques		
Ashing	Peat	Dugmore (1989); Pilcher and Hall (1992)
Acid digestion	Peat	Dugmore *et al*. (1995)
Microwave digestion	Peat	Payne and Blackford (2006)
Alkali treatment	Diatom-rich lacustrine sediment	Rose *et al*. (1996)
Density separation	Mineral-rich, e.g. lacustrine, marine, archaeological, soil	Turney (1998); Abbott *et al*. (2011); Lane *et al*. (2014)
Melting & centrifugation	Ice	Davies *et al*. (2010b); Abbott and Davies (2012)
Magnetic separation	Mineral-rich	Mackie *et al*. (2002); Griggs *et al*. (2014b)
X-ray diffraction	Marine	Andrews *et al*. (2006)
Thin sections & micromorphology	Lacustrine (especially varve sequences); marine; peat	Wulf (2004); De *et al*. (2008); De Vleeschouwer *et al*. (2008); Griggs *et al*. (2014b)
Non-destructive techniques		
Continuously imaging flow cytometer	Lacustrine	D’Anjou *et al*. (2014)
XRF	Lacustrine, marine, peat	Kylander *et al*. (2012)
Magnetic susceptibility	Peat & mineral-rich sediment	Peters *et al*. (2010)
Spectrometry (light reflectance)	Peat	Caseldine *et al*. (1999)
X-radiography	Peat	Dugmore and Newton (1992)

A new non-destructive method, developed by D’Anjou *et al.* (2014), based on a fluid-imaging flow cytometer approach has been successfully applied to lacustrine material and has much to offer other sedimentary records. Other non-destructive techniques, such as X-ray fluorescence (XRF), magnetic susceptibility and light reflectance spectrometry, are all rapid scanning techniques that highlight specific depths that warrant further investigation. Their success in a cryptotephra context is somewhat inconsistent and will depend largely on the contrast between the composition of the host material and the chemistry and concentrations of the glass shards (e.g. Gehrels *et al.*, 2008; Kylander *et al.*, 2012; Wulf, 2013). If the contrast is optimized for the record under scrutiny, then these techniques have some potential for the identification of sampling targets, especially for long core sequences.

One notable growth area in recent years has been the search for cryptotephras within marine sequences. Although macroscopic tephras in the marine realm have been reported for many years, it is only very recently that the hidden and often fine-grained fraction that predominantly makes up the cryptotephra record has been investigated (e.g. Bourne *et al.*, 2010; Brendryen *et al.*, 2010; Abbott *et al.*, 2013, 2011; Cage *et al.*, 2011; Jennings *et al.*, 2014). This work, of course, is not without challenges, but is crucial if we are to establish three-way correlations between the continental, marine and ice-core records to explore the climatic phase relationships between these components. A Geological Society Special Publication (No. 398) has been dedicated to capturing recent work in the marine realm (Austin *et al.*, 2014; Lowe, 2014).

### Geochemical fingerprinting cryptotephra deposits

#### Major element analysis

Once a cryptotephra deposit has been identified, one of the most challenging steps is to geochemically characterize the major element composition of the shards by electron microprobe. Since its development in the late 1960 s (Smith and Westgate, 1968), single-shard electron microprobe analysis (EMPA) has been routinely applied to acquire a geochemical fingerprint for tephra and cryptotephra deposits. Single-shard analyses are now fundamental for tephra work to provide a glass composition unaffected by any lithic or crystalline material that may be represented in the analysis of a bulk sample. In the early days of UK cryptotephra work, a key influential figure was Dr Peter G. Hill (1942–2010) who provided training and inspiration for all users of the electron microprobe at the University of Edinburgh (Hunt, 2011). His boundless enthusiasm and support proved crucial to ensure the acquisition of robust geochemical data from small and thin glass shards.

Wavelength dispersive spectrometry is the preferred method but some laboratories have successfully reported major element results acquired via energy-dispersive spectrometry or a combination of the two (e.g. Coulter *et al.*, 2010). Wavelength dispersive spectrometry normally provides 10 major elements as oxides (SiO_2_, Al_2_O_3_, FeO, TiO_2_, MnO, MgO, CaO, Na_2_O, K_2_O, P_2_O_5_) and each laboratory will now typically follow analytical set-ups that are bespoke for tephra analysis that employ a narrow beam size and minimize alkali mobilization (Hayward, 2012). Over the years, analytical limitations have been overcome and operating conditions have been optimized to allow shards as small as 10 μm to be successfully analysed (e.g. Hunt and Hill, 1993; Hayward, 2012; Pearce *et al.*, 2014). Inter-laboratory comparison exercises also ensure the highest levels of precision and accuracy and make recommendations for systematic operating conditions and the reporting of results (Kuehn *et al.*, 2011). What is challenging, however, is ensuring that just a few microscopic glass shards are prepared on a flat, resin-embedded and polished surface for microprobe analysis. This can often feel like an arduous and somewhat frustrating undertaking, especially if the deposit contains just a few shards. Several laboratories now use micro-manipulation techniques to individually pick the shards and both slides and resin blocks are used for securing the shards to facilitate their polishing (e.g. Kuehn and Froese, 2010; Lowe, 2011; Hall and Hayward, 2014). The final polishing stages require care to ensure that the small number of precious shards are not removed inadvertently. Again, the key for successful execution of cryptotephra work is a large spoonful of patience. Kuehn and Froese (2010) and Hall and Hayward (2014) provide very helpful advice to guarantee success when preparing samples for microbeam analysis.

Without good quality major element data, tephra correlations between widely separated records would be impossible. Gone are the days of providing a best guess for the identification of a tephra based solely on age estimates and stratigraphic positions. Distal tephrostratigraphy is far more complex than previously thought and long-distance transport of tephra now requires the analyst to expand the search beyond the nearest and, perhaps, the most obvious volcanic source. As trachytic tephras from Mediterranean sources are rapidly encroaching on the Icelandic dispersal areas (Lane *et al.*, 2011a) and Alaskan tephras are appearing within European sequences (Jensen *et al.*, 2014), robust geochemical data, together with age and stratigraphical information, underpin such noteworthy discoveries. Essential to this work is the comparative compositional analysis (via electron micro-probe analysis) of proximal and distal deposits to establish volcanic provenance. Such comparisons become more complex as we delve further back in time due to the discontinuous proximal records. The volcanic histories and compilations that are available to us from the near-field records, however, are invaluable (e.g. Larsen *et al.*, 2001, 1999; Gudmundsdóttir *et al.*, 2011a,2011b; Óladóttir *et al.*, 2011a,^2012^, 2011b) and highlight the noteworthy contributions of Gudrun Larsen (a student of Thórarinsson) and her colleagues. In addition, geochemical comparisons of proximal and distal deposits have been instrumental in demonstrating that fine-grained shards retain their signatures in a range of depositional environments (Dugmore *et al.*, 1992). Others, however, have suggested that the chemical stability of tephras is significantly influenced by their composition (Pollard *et al.*, 2003).

One of the most recent confounding issues is the discovery of several tephras that are close in age and major element geochemistry (e.g. Lane *et al.*, 2012b; Bourne *et al.*, 2013). Consequently, some time periods and palaeorecords will be plagued by difficulties if they contain a series of tephras that lack a unique geochemical fingerprint. Employing these deposits as isochrons will be challenging, especially if a potential correlative proxy record contains only one or two of these indistinguishable deposits. In such cases, careful scrutinization of compositional data is crucial and Bourne *et al.* (2015) demonstrate how subtle differences in minor elements, e.g. TiO_2_, can be very informative as discriminatory tools. It goes without saying therefore that a prerequisite of this work is robust and precise geochemical data within a sound stratigraphic framework.

#### Trace element analysis

In addition to characterizing the major element signature of cryptotephra deposits, recent years have also seen an upsurge in the analysis of trace elements. Initial experimentation focused on the analysis of bulk samples (e.g. Pearce *et al.*, 2004) and by now single shards between 10 and 20 μm in diameter can be successfully analysed (e.g. Pearce *et al.*, 2007, 2011; Tomlinson *et al.*, 2010; Abbott *et al.*, 2014). Analysing such small shards does lead to some challenges and potential future directions are outlined by Nick Pearce (Pearce, 2014; Pearce *et al.*, 2014). It is worth noting that this technique is not pursued as an alternative but as a supplement to the major element signatures in search of a diagnostic fingerprint. Trace element analysis by laser ablation-inductively coupled plasma-mass spectrometry offers considerable value as an additional discrimination tool. For instance, Albert *et al.* (2012) highlight the added value of obtaining a full complement of major and trace elements to confidently underpin a marine–terrestrial correlation of tephra deposits in the Aeolian Islands (south Tyrrhenian Sea). Other examples demonstrate how subtle variations in trace elements can disclose the preservation of different evolutionary phases of an eruptive event (Abbott *et al.*, 2014) and how miscorrelations may arise if only major element results are available (Davies *et al.*, 2014). Questions have also been raised with regard to the isochronous nature of various Saksunarvatn Ash deposits found in northern Europe following the acquisition of trace elements (Davies *et al.*, 2012; Bramham-Law *et al.*, 2013). Trace element data also have much to offer as a discrimination tool for tephras that are close in age and exhibit a similar major element chemical composition. Investigations by Allan *et al.* (2008) in the south-west Pacific demonstrate that tephras with similar major-element composition were easily distinguishable with respect to trace elements (Lowe and Alloway, 2014). In Europe, however, trace element signatures for Icelandic tephras that are close in age have tended to support their common origin rather than allowing their discrimination (e.g. Lane *et al.*, 2012b). As instrumental conditions are now optimized for analysing small shards, it is now timely to explore distal sets of compositionally similar tephras in search of unique fingerprints.

#### Data comparison and statistical tools

Once both major and trace element data have been acquired, statistical tools and data comparison methods allow a tephra correlation and volcanic provenance to be tested. A contentious issue for tephrochronologists is whether to normalize the major element data to 100% for comparative purposes. Some advocate that normalization is necessary to remove the water content of the glass (acquired from magmatic water and/or in the post-depositional environment), whereas others believe that normalization may mask the quality of the data (Hunt and Hill, 1993; Pollard *et al.*, 2006; Pearce *et al.*, 2014, 2008b). This is a key consideration at the outset of any data comparison and correlation exercise. Whatever the approach, the non-normalized analytical total should always be presented so the data can be re-calculated and secondary standard analyses should also be given to assess the quality of the data-set (Pearce *et al.*, 2014).

In its simplest form, most analysts would explore graphical representations such as bivariate and triangular plots in search of overlapping compositions (or otherwise). A wide range of statistical tools are also employed, including tests of similarity (similarity coefficient: Borchardt *et al.*, 1972), difference (statistical distance: Perkins *et al.*, 1995; Pearce *et al.*, 2008a) and the use of multivariate analysis (principal-component analysis: Pollard *et al.*, 2006; discriminant function analysis: Tryon *et al.*, 2010). For further details on statistical techniques employed in tephra studies, see, for example, Lowe *et al.* (2015).

A key element of data comparison methods is the archiving of compositional data in accessible databases. One of the earliest databases designed specifically for the cryptotephra analyst was Tephrabase (www.tephrabase.org), launched online in 1995 (Newton, 1996; Newton *et al.*, 2007). Not only does this act as a repository, but it also functions as a tool whereby several different search options can be selected. This particular database includes compositional data for Icelandic, Mexican and Eifel-sourced tephras (Newton *et al.*, 2007; Riede *et al.*, 2011). Other databases are also available (e.g. RESET (Bronk Ramsey *et al.*, 2015b), PANGAEA: www.pangaea.de, PETLAB: http://pet.gns.cri.nz) and there is momentum within the community to develop a global database that will significantly improve and facilitate the data comparison exercises (Kuehn *et al.*, 2013). Such a development is clearly desirable as the geographical distribution of ash fallout never ceases to remain static.

## Taphonomic processes

**Figure 3:**
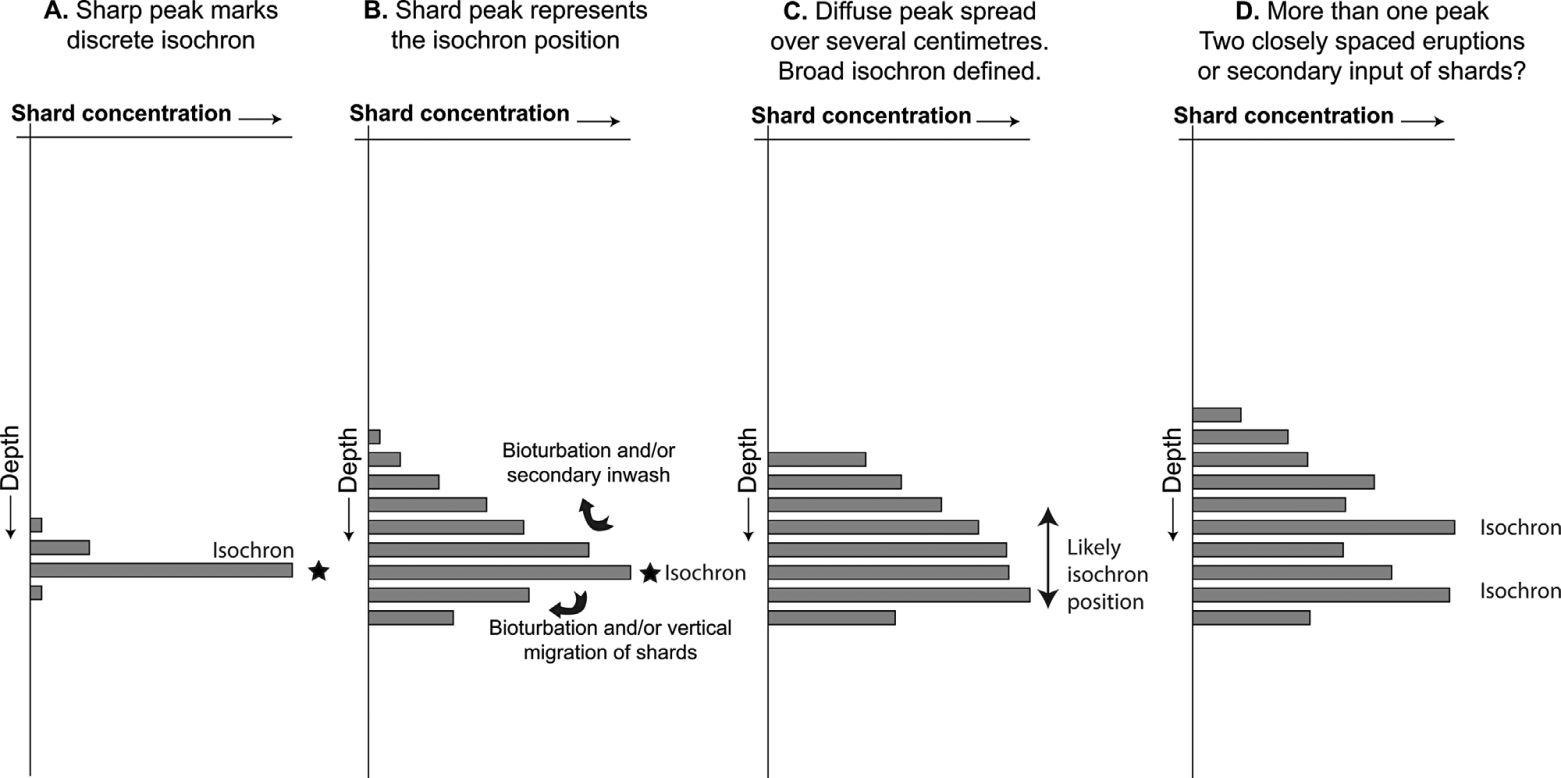
Schematic illustration of possible shard distribution profiles that may be observed in association with cryptotephra deposits. Indicative examples of where the isochron may be placed are shown.

In addition to the geochemical fingerprinting, an examination of taphonomic processes in a cryptotephra study is paramount. When exploring visible tephras, several clues may indicate the influence of post-depositional reworking, for example irregularities in grain size and the thickness of the deposit (e.g. Lowe, 2011). For studies involving cryptotephras, however, the clues are often indistinct and difficult to decipher. With no macro-features visible in the core or outcrop, the glass shards of the cryptotephra deposit are often the only indicators of real value. The shape of a shard concentration profile, geochemical inconsistencies, grain-size morphologies and vertical distribution within a sequence are among some of the key indicators within a sedimentary environment (e.g. Gudmundsdóttir *et al.*, 2011a,2011b; Griggs *et al.*, 2014b). While these are primarily used to define the position of the isochron, they may perhaps also reveal signs of post-depositional reworking that may impair the veracity of the cryptotephra as an isochron. Cryptotephras formed entirely from primary fallout are clearly marked by a discrete horizon of glass shards indicative of a rapid input of ash (Fig. 3A) (e.g. Lane *et al.*, 2013a). In other cases, a peak in shard concentration can be deciphered, but evidence of bioturbation and/or secondary inwash is also noted (e.g. Fig. 3B). Within some records, however, a distinct peak is not always preserved and shards may be broadly scattered within a vertical profile (Fig. 3C,D) (e.g. Payne and Gehrels, 2010; Davies *et al.*, 2012). Although such features bring unwanted complexities to the quest for uncomplicated and prevalent tie-points, these observations can also reveal a wealth of information on the processes operating within and around the depositional environment at the time of, and after, tephra deposition.

Cryptotephra features may allow insight into taphonomic, sedimentary, anthropogenic and geomorphological processes. For instance, Pyne-O’Donnell (2010) showed how catchment inlets significantly influence the distribution and sedimentation of cryptotephras in a lake basin which varied according to the geochemical composition of the deposit. Davies *et al.* (2007) inferred that glass shard input into a high mountain lake may be prolonged as late Holocene perennial snow-beds act as traps higher up in the catchment. Bergman *et al*. (2004) also implied that a patchy tephra distribution across a peat surface may be due to the effects of snow cover at the time of eruption. Swindles *et al.* (2013) argued that human activity by way of burning and peat erosion during the mid-Holocene should not be underestimated as a significant agent in re-mobilizing tephras deposited in the landscape while other studies, rather worryingly, imply that tephras are prone to density settling through soft sediment (Beierle and Bond, 2002). Even peat bog environments, where mixing and movement are thought to be negligible, are prone to vertical migration of glass shards (Payne and Gehrels, 2010).

In the marine environment, a complex suite of processes may disturb the preservation of a discrete cryptotephra horizon as well as impart a delay in its transport and deposition (e.g. Brendryen *et al.*, 2010, 2011). High inputs of ice-rafted detritus concurrent with elevated abundances of glass shards would strongly suggest that ice rafting is the primary transport mode. In such cases, a delay in tephra deposition is anticipated due to the storage of tephra in ice sheets before calving (Brendryen *et al.*, 2010; Griggs *et al.*, 2014b; Kuhs *et al.*, 2014). A geochemically heterogeneous tephra would also indicate this secondary transport process as tephras from several eruptions may be amalgamated within the ice. A broad or dispersed zone of glass shards in a marine profile (e.g. Fig. 3D) may also indicate the degree of mixing by bioturbation (e.g. Todd *et al.*, 2014), but this feature may also indicate the regular input of ash from closely spaced eruptions that cannot be resolved in a slowly accumulating depositional environment (e.g. Bourne *et al.*, 2013). Griggs *et al.* (2014a) have tantalizingly demonstrated how the sedimentary processes associated with tephra deposition in the marine environment can be visualized and demarcated in three dimensions via an X-ray microtomography technique. This approach has much to offer in the future for defining the true placement of isochrons and for employing tephras as tracers to gain insight into taphonomic processes. Indicators of reworking have also been identified by microfacies analysis and micro-XRF core scanning techniques (e.g. Wutke *et al.*, 2015).

Although features of post-depositional processes can be catastrophic for those searching for isochrons, there is also much to be gained from adopting the ‘total tephrochronology’ approach of Dugmore and Newton (2012). To date, this approach has been applied to visible tephras in proximal settings, giving considerable insight into landscape development, archaeological change and geomorphological processes such as cryoturbation and soil erosion (Streeter and Dugmore, 2013a,2013b). A multitude of stratigraphic sections, including those that are poorly developed, are required to optimize this large-scale approach. Whether cryptotephra studies can offer such detailed insight into landscape-scale processes remains to be seen, but processes at this scale should not be overlooked or decoupled from the micro-scale features of a cryptotephra deposit.

## Building tephrochronological frameworks

As well as extending the geographical distribution of tephra fall-out, cryptotephra investigations have also identified several new, previously unknown tephra deposits in distal settings. These discoveries augment the volcanic history and dispersal compilations derived from the proximal realm (e.g. Larsen *et al.*, 2001, 1999; Thordarson and Larsen, 2007; Larsen and Eiríksson, 2008b,b; Thordarson and Höskuldsson, 2008; Óladóttir *et al.*, 2011a,2011b). What has arisen is a new task of building comprehensive frameworks, lattices and catalogues of volcanic events (e.g. Haflidason *et al.*, 2000; Davies *et al.*, 2002; Hall and Pilcher, 2002; Wulf, 2004; Óladóttir *et al.*, 2008; Smith *et al.*, 2011; Zanchetta *et al.*, 2011; Blockley *et al.*, 2012; Wulf *et al.*, 2012; Bourne *et al.*, 2015). These publications outline a synthesis of known eruptive events, their respective geochemical compositions and best age estimates within a given time interval and represent a prerequisite for tephrochronological work. Geochemical signatures, stratigraphic positioning and age estimates can be considered as the essential backbone or building blocks for these frameworks (Lowe *et al.*, 2008).

**Figure 4:**
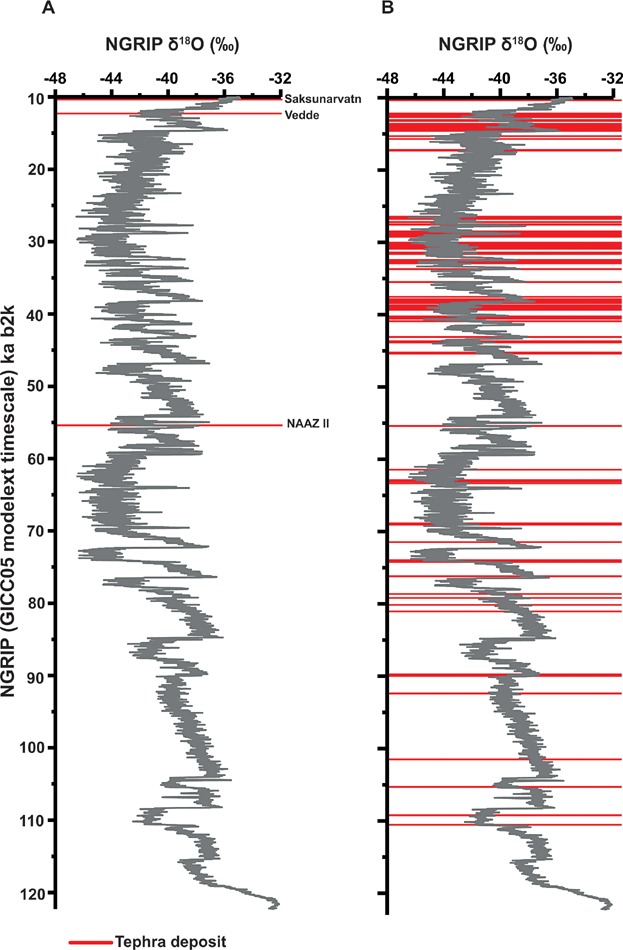
Tephrostratigraphy of the Greenland ice-core records plotted against the NGRIP oxygen isotope stratigraphy from Svensson *et al.* (2008) and Wolff *et al.* (2010). (A) Tephra deposits identified within the Summit cores (GRIP & GISP2) by Grönvold *et al.* (1995) and Zielinski *et al.* (1997) with a predominant focus on visible deposits. (B) Cryptotephra deposits identified to date within the NGRIP, GRIP and NEEM ice cores (Mortensen *et al.*, 2005; Davies *et al.*, 2008 2010b, 2014; Abbott *et al.*, 2012; Bourne *et al.*, 2015, 2013). NEEM and GRIP tephras are shown on the NGRIP record according to a timescale transfer and synchronization (Rasmussen *et al.*, 2013; Seierstad *et al.*, 2014). This figure is available in colour online at wileyonlinelibrary.com.

The importance of frameworks is highlighted by the incredible number of new cryptotephra discoveries. Dugmore (1989) mentioned that British peat bogs may contain evidence of 12 of the key Holocene eruptions that were known to have been dispersed beyond Iceland. These have, indeed, been identified together with a further 11 cryptotephra deposits that are only known from far-field occurrences (Fig. 2). In a recent compilation, Lawson *et al.* (2012) mentioned that a further 84 Holocene cryptotephra deposits have been found but are only reported from one or two sites. New tephra discoveries have also been gleaned from medial and proximal settings (e.g. Gudmundsdóttir *et al.*, 2012). Similarly for the Lateglacial period in Europe, only three key tephras were known based on work reported in the 1990 s – the Laacher See Tephra (12 880 varve a BP: Brauer *et al.*, 1999), the Vedde Ash (12 171 ± 114 b2k: Rasmussen *et al.*, 2006) and the Saksunarvatn Ash (10 347 ± 89 b2k: Rasmussen *et al.*, 2006). By now, at least 15 additional tephras have been identified in distal settings, providing invaluable information on the Icelandic volcanic history during this period (e.g. Lowe *et al.*, 2008; Lind and Wastegård, 2011; Lind *et al.*, 2013; Blockley *et al.*, 2014). Without a doubt, medial and distal sites have a clear role to play in reconstructing volcanic histories when proximal records may be incomplete (e.g. Lowe, 2011). The story continues into the last glacial period with new cryptotephra results from Greenland ice cores providing an exceptional catalogue of volcanic events (Fig. 4) (Mortensen *et al.*, 2005; Davies *et al.*, 2014, 2010b; Abbott *et al.*, 2012; Abbott and Davies, 2012; Bourne *et al.*, 2015). Over 100 tephras have now been reported from pre-Holocene ice, a significant increase from the three tephras reported in the mid 1990 s (Grönvold *et al.*, 1995; Zielinski *et al.*, 1997).

Given the scale of new tephra and cryptotephra discoveries and the complexities that we now face, framework papers are crucial to avoid any potential miscorrelations that may arise because of an incomplete volcanic event stratigraphy (e.g. compare Davies *et al.*, 2010b with Bourne *et al.*, 2013). Indeed, Antony Long, Editor-in-Chief of *Journal of Quaternary Science* (JQS) nominated Haflidason *et al.* (2000) as one of the most influential papers published in that journal (Long, 2013). Haflidason and colleagues outlined one of the first frameworks of Icelandic tephra deposits encompassing the last 400 ka. It is also worth noting that JQS also became and continues to be the journal of choice for a number of tephra publications, in part stemming from founding editor John Lowe’s desire to build a journal of global reach. Examples from very early issues of JQS include Thompson *et al.* (1986) and Lowe (1988). Moreover, during his editorship in the early 2000 s, James Scourse often made claims that the journal’s name would be more appropriate as the ‘Journal of Tephrochronology Studies’. Such comments reflect the continued impact of tephrochronology on wide-ranging Quaternary and archaeological studies.

As distal sites yield a complex record of previously unknown volcanic events, such frameworks are regularly refined and revised (e.g. compare Davies *et al.*, 2002 with Blockley *et al.*, 2012) and also inform studies of eruptive history and hazard assessment (see ‘Eruptive history and hazard assessment’ below). Before updating a framework, however, any new tephra discovery requires a careful assessment of origin, in particular to rule out any potential secondary processes that may have re-deposited the glass shards (see ‘Taphonomic processes’ above). Some protocols have been developed that help to assess the integrity of cryptotephra deposits within the marine environment and assess their value as potential isochrons (Griggs *et al.*, 2014b). Future work may need to consider a similar toolkit for those working in the terrestrial realms.

## Cryptotephra applications

**Figure 5:**
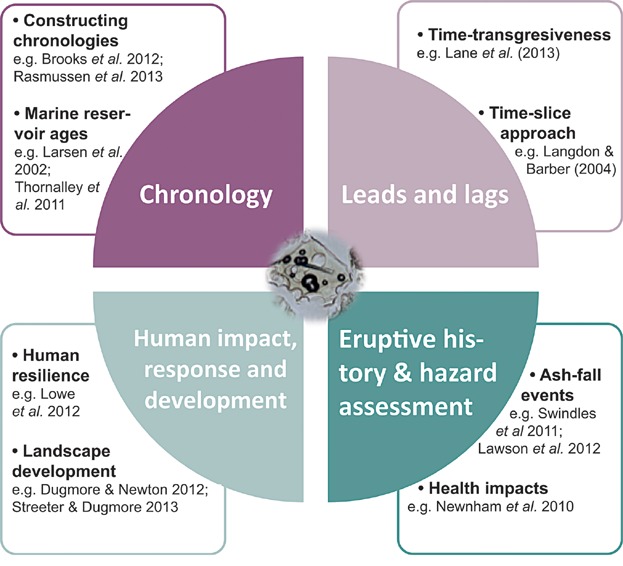
Synthesis of the four main applications of cryptotephra studies, including indicative references. This figure is available in colour online at wileyonlinelibrary.com.

Over the last 50 years, tephrochronology has been applied to address a whole range of scientific questions from palaeoseismology to environmental reconstructions (e.g. see Lowe, 2011). The advent of cryptotephra research in Europe has widened the possibilities, allowing far-reaching objectives to be tackled in novel geographical areas. These are summarized as four key themes in Fig. 5 and will be discussed in turn below.

### Building and improving chronological models

Where an age has been assigned to a tephra deposit, this age is particularly valuable for building and improving chronological models via tephrochronology. Tephra age estimates can form the sole basis of a model (e.g. Brooks *et al.*, 2012; Gudmundsdóttir *et al.*, 2012) or can be imported into a chronological model to provide a fix point that may reduce the age uncertainties based on other dating techniques (e.g. Wastegård and Davies, 2009; Housley *et al.*, 2010; Lane et al., 2011b,c; Rosqvist *et al.*, 2013). Alternatively, tephra age estimates can be used to test the model constructed by other dating techniques (e.g. Brauer *et al.*, 2014). These applications depend entirely on the quality of the age obtained for a tephra deposit using tephrochronometry. Ages can be derived via direct (e.g. fission track or K/Ar methods) or indirect methods whereby an age is assigned to the encompassing host material. An age for the latter may be obtained by high-precision radiocarbon dating or be based on the stratigraphic position of a tephra within incremental records, such as varve or ice-core records (e.g. Zillén *et al.*, 2002; Wulf, 2004; Svensson *et al.*, 2008; Dörfler *et al.*, 2012; Bourne *et al.*, 2015) (see also Lowe, 2011 and Lowe and Alloway, 2014 for further details on dating tephra deposits). One particular noteworthy development is the increased adoption of a Bayesian modelling approach to improve and refine the best age estimate for a tephra deposit that is bracketed by a series of high-precision radiocarbon ages. These have been particularly important for both well-known and newly identified cryptotephras that are not preserved within annually resolved records. For instance, Wohlfarth *et al.* (2006) and Matthews *et al.* (2011) provided new age estimates for Lateglacial and early Holocene tephras, such as the Borrobol, Penifiler, Hässeldalen and Askja tephras found in Scottish and Swedish records. Olsen *et al.* (2010) adopted a similar approach to provide a well-constrained age of 6668–6653 cal a BP for the Mjáuvøtn tephra. Further south, Blockley *et al.* (2008), Albert *et al.* (2015) and Bronk Ramsey *et al.* (2015a) refined the age constraints for key Mediterranean tephras. The same approach is also particularly valuable for comparing tephra age estimates derived from two independent timescales, such as the Greenland ice-core chronology (GICC05) and the INTCAL calibration curves (e.g. Lohne *et al.*, 2013, 2014). Coeval tephra deposits traced between different ice cores can also supplement a chemostratigraphic matching approach for synchronizing Greenland records to a master chronology (Rasmussen *et al.*, 2013; Seierstad *et al.*, 2014).

Unquestionably, successful use of tephra deposits for building and improving chronologies depends entirely on the characterization and correct identification of the source event. Evidently, a miscorrelation and importing the wrong age estimate would introduce significant errors into the chronological model. For instance, Albert *et al.* (2015) demonstrated that the Y-3 proximal type site in the Ionian Sea has been incorrectly identified and, hence, Ar−Ar ages from this deposit should no longer be employed as the age of this important isochron.

A further benefit of tephrochronology is the opportunity presented to constrain marine reservoir ages. Several studies have successfully constrained the changes in the marine radiocarbon reservoir in different periods showing complex spatial and temporal variations (e.g. Austin *et al.*, 1995; Bondevik *et al.*, 2001; Waelbroeck *et al.*, 2001; Larsen et al., 2002; Eiríksson *et al.*, 2004; Austin *et al.*, 2011; Lowe *et al.*, 2013). For instance, Thornalley *et al.* (2011) observed reservoir age estimates of up to 2000 years south of Iceland during Heinrich Stadial 1 and the Younger Dryas, and Eiríksson *et al.* (2004) reported reservoir ages up to 450 years during the Late Holocene on the North Icelandic Shelf. These investigations have largely focused on well-defined and visible horizons. Very few studies, however, have explored the use of cryptotephra deposits for constraining the reservoir changes (e.g. Austin and Hibbert, 2012). With significant developments in the identification of discrete cryptotephra deposits in the marine environment, there is considerable scope to employ these horizons to constrain the reservoir changes during the glacial period assuming that an independent or terrestrial-based calendrical age estimate is also available.

### Constraining leads and lags in the climate system

A key motivation for cryptotephra work in recent years has been the quest for constraining rapid climatic events and to integrate disparate palaeoclimatic records to assess the degree of climatic synchroneity between different components of the climate system. A landmark paper that exemplifies what tephrochronology has to offer was recently presented by Lane *et al.* (2013a). By tracing the Vedde Ash in cryptotephra form within the varved record of Meerfelder Maar, Lane *et al.* (2013a) demonstrated the time-transgressive nature of climatic changes during the Younger Dryas in NW Europe. Climatic amelioration during the Younger Dryas linked to the resumption of the thermohaline circulation was constrained to 100 years before the Vedde Ash in Germany, but 20 years after this Vedde Ash isochron in western Norway. Such well-defined and widespread tephra deposits preserved within annually resolved records are somewhat rare but incredibly valuable and demonstrate the opportunities for adopting a differential dating approach to constrain the time between tephra deposition and a rapid climatic event. Wulf *et al.* (2013) adopted a similar approach to identify a 200-year interval between the Laacher See eruption and the start of the Younger Dryas in varved records along a transect in north central Europe. This synchronous response is well constrained and highlights the powerful approach of combining tephra isochrons with annually resolved records. A study by Macleod *et al.* (2014) also demonstrates how the Vedde Ash is preserved within a floating varve sequence from Sweden. A further advance may well emerge in the future where rapid climate events preserved within ice and a varved depositional environment are bracketed by two or more common tephra isochrons. As more and more new tephras are identified in the Greenland ice-core records in close association with abrupt climatic transitions, this is a realistic prospect (Davies *et al.*, 2014; Bourne *et al.*, 2015).

Where annually resolved records are unavailable, however, tephra or cryptotephra deposits continue to represent powerful tie-points during an assortment of time periods. Wastegård *et al.* (2005); for example, employed the widespread 5e-Eem/RHY-1 to constrain the oceanic and continental response in the North Atlantic during the Eemian climatic optimum. Although synchronous changes were apparent in the Norwegian Sea area, pollen stratigraphic changes suggested that these lagged behind the climatic optimum experienced in continental Europe. During the last glacial period, Austin *et al.* (2004) proposed that climatic shifts during Greenland Interstadial-15 occurred synchronously between the atmospheric and oceanic realm with ice and marine records tied according to the position of the North Atlantic Ash Zone II. A further correlation by Austin *et al.* (2012); based on the Faroe Marine Ash Zone II deposited during Greenland Stadial-3, also illustrates the complexities of millennial-scale climatic events in the North Atlantic Ocean that are not imprinted on the Greenland ice cores. Such interpretations would be difficult without the tephra isochron to provide the chronostratigraphic constraint. Finally, asynchronous changes are identified by Langdon and Barber (2004) during the mid-Holocene period, whereby seven Scottish peat bogs are anchored by the Glen Garry and Hekla-4 tephras. Bog surface wetness indices from these sites are presented on a time-slice reconstruction for each tephra deposit. Tephrochronology and cryptotephrochronology offer much promise for facilitating a time-slice approach in a range of different time periods, but depend entirely on tracing common tephras and cryptotephras in a range of well-characterized proxy records.

### Eruptive history and hazard assessment

Although the advent of cryptotephra research has revealed several new previously unknown volcanic events, their significance in terms of eruptive history and hazard assessment was somewhat unclear until the extraordinary event of the Eyjafjallajökull 2010 ash cloud (Davies *et al.*, 2010a; Gudmundsson *et al.*, 2012). The unfolding of the Eyjafjallajökull events has given distal cryptotephra studies significant weight and authority in terms of informing ash-fall frequencies in the past (Bourne *et al.*, 2010; Swindles *et al.*, 2011; Lawson *et al.*, 2012). Moreover, with recent reports of transatlantic tephra transport, European aviation authorities may well need to cast their eyes beyond the usual volcanic suspects (Jensen *et al.*, 2014). Such studies also support reconstructions of past atmospheric circulation changes (e.g. Lacasse, 2001; Lacasse and van den Bogaard, 2002) and deposition and dispersal mechanisms (e.g. Stevenson *et al.*, 2013, 2012, 2015). In New Zealand, cryptotephra investigations are also gathering pace to inform hazard assessment (Gehrels *et al.*, 2006). Executing cryptotephra studies in such active volcanic areas can, however, be confounded by difficulties, particularly if redeposited ash may be difficult to identify (Gehrels *et al.*, 2006). There is also scope for cryptotephra investigations to inform studies on the potential health impacts of fine ash particles deposited in distal settings (e.g. Newnham *et al.*, 2010).

### Human impact, response and development

The use of tephrochronology in archaeological settings can be traced back to Thorarinsson’s work in the 1940 s and 1950 s, although an earlier example dating to 1931 in New Zealand (Oliver, 1931) was described by Lowe (1990). Iceland, in particular, has a rich history of investigations that have exploited volcanic event horizons as a tool to date farm ruins and thus to delimit settlement occupation and abandonment (e.g. Thórarinsson, 1944, 1981a and references therein; Dugmore *et al.*, 2007). The potential of employing cryptotephra studies in archaeological settings is, however, somewhat underutilized. Nonetheless, several success stories published in recent years typify what can be achieved. For instance, Hannon and Bradshaw (2000) made use of the Landnám tephra, preserved as a cryptotephra deposit, alongside radiocarbon ages to argue that the timing of human settlement on the Faroe Islands dates back to the 6th century AD. The Microlite tephra provides an anchor in the age model developed by Plunkett *et al*. (2009) in the multi-proxy investigation of an Irish peat bog within which an Iron Age body was discovered. In addition, Balascio *et al.* (2011) illustrated how the preservation of at least two cryptotephra deposits within an Iron Age/Viking Site in the Lofoten helped to constrain the age of boat houses at this site. Interestingly, one of the tephras found at this particular site is the AD 860B deposit commonly found in Ireland (Hall and Pilcher, 2002), and recently revealed to be the Alaskan White River Ash (Jensen *et al.*, 2014). Transatlantic comparisons, not only in terms of climatic contrasts, but also of cultural history and development are now possible.

Noteworthy accomplishments have also been achieved by the RESET^2^ project. Systematic application of cryptotephra techniques by RESET project members at a wide range of sites throughout Europe has brought this dating technique to the forefront of archaeological studies (e.g. see Lane *et al.*, 2014 and references therein; Housley and Gamble, 2015). Investigations by this research consortium demonstrated that discrete cryptotephras are preserved within cave deposits, rock-shelters as well as open-air archaeological sites (e.g. Borić *et al.*, 2012; Lowe *et al.*, 2012; Douka *et al.*, 2014; Housley and Gamble, 2015). Optimal conditions for cryptotephra preservation are often difficult to foresee with several negative results reported, but the best return seems to be at sites with large cave openings, low-energy environments where the bioturbation and erosion processes are low, and often within ‘off-site’ contexts, typically lakes or peat bogs, that can be correlated to occupation sites (Lane *et al.*, 2014; Housley and Gamble, 2015). Riede and Thastrup (2013) strongly argued that future studies should adopt a transect strategy whereby archaeological sites as well as nearby lakes or peat bogs should be sampled to fully assess the continuity of cryptotephra deposits.

These recent exemplary studies mark the beginning of an up-and-coming area and Riede and Thastrup (2013) in particular, suitably promote the exciting opportunities that lie ahead. Indeed, one of the key outputs of the RESET project is a classic illustration of the resilience of Neanderthals and early modern humans (Lowe *et al.*, 2012). The large Campanian Ignimbrite eruption and the abrupt climate changes at that time failed to impact or generate a significant response from these populations (Lowe *et al.*, 2012). Without the identification of hidden Campanian Ignimbrite deposits to anchor the cave sites and long core sequences in focus, such an interpretation would not have been possible.

Although only applied in proximal settings, another emergent application of tephrochronology is landscape development, especially with respect to understanding geomorphological processes (e.g. soil erosion, periglacial processes) as well as human–environment interactions (e.g. Dugmore *et al.*, 2009; Dugmore and Newton, 2012; Streeter and Dugmore, 2014,2013a, 2013b). A further facet of this work is the insight provided by micro-scale tephra deposits (both spatial patterning and thickness variations across a landscape) for the identification and anticipation of critical transformations in land surfaces (Streeter and Dugmore, 2013a). Such studies have wide-ranging implications for assessing resilience, sustainability science and ecosystem services.

## Future directions and challenges

Without doubt, the emergence of cryptotephras as key correlation and dating tools has been revolutionary. Isochron maps are continually redrawn to reflect the rapid rise in cryptotephra discoveries and regional tephra frameworks are perpetually updated as a full picture of volcanic events and associated deposits emerge. As yet, the biggest impact of these studies has been felt in Europe and the North Atlantic region, but new investigations are gathering pace in East Africa (e.g. Lane *et al.*, 2013b), north-eastern Russia (e.g. Ponomareva *et al.*, 2013), New Zealand (e.g. Gehrels *et al.*, 2006; Holt *et al.*, 2011), South America (e.g. Wastegård *et al.*, 2013), North America (e.g. Pyne-O’Donnell *et al.*, 2012) and Antarctica (e.g. Dunbar and Kurbatov, 2011; Narcisi *et al.*, 2012). This research area has given rise to a new generation of tephrochronologists who show considerable potential to maintain the upward trajectory of this technique.

We are, of course, faced by many challenges, particularly in relation to taphonomic and post-depositional processes with several illustrations of unpredictable and puzzling distribution patterns (e.g. Davies *et al.*, 2001; Pyne-O’Donnell, 2010). Future studies may well need to routinely investigate more than one core within depositional sites to fully explore the variable nature of cryptotephra distribution (e.g. Pyne-O’Donnell, 2010; Dugmore and Newton, 2012; Riede and Thastrup, 2013). Not only will this work have implications for understanding the depositional processes, but it also lays the foundation for correctly defining the stratigraphic position of a cryptotephra deposit. Visualization techniques, on both a macro-scale (e.g. high-resolution photogrammetric techniques; Streeter and Dugmore, 2014) and a micro-scale (e.g. X-ray microtomography; e.g. Griggs *et al.*, 2014a), offer considerable promise in this respect.

As additional tephras/cryptotephras are added to chronostratigraphic frameworks, complexities arise and bring with them new challenges. In particular, the occurrence of compositionally similar tephras within a short time interval presents several challenges. In such cases, some tephras will inevitably offer more value than others as widespread time-parallel marker horizons (e.g. Davies *et al.*, 2012; Bourne *et al.*, 2015). Defining the relative value of a tephra deposit will help inform sampling work in other records and a key priority for future work is an endeavour to finalize the tephra frameworks to allow unambiguous application of this technique. Whether this is possible remains to be seen and resolving compositional and stratigraphical ambiguities is of utmost importance. Further testing the value of trace element analysis to give rise to diagnostic geochemical fingerprints will be particularly fascinating. Characterizing trace element signatures within proximal tephra deposits to capture the full trends derived from magma mixing, mingling or magmatic evolution is also a priority. More often than not, a distal network of palaeorecords may preserve different phases of the same volcanic event (Abbott *et al.*, 2014), resulting in a multiple fingerprint scenario outlined by Lowe (2011). Assessing whether they belong to the same event can only be compared if proximal stratigraphies are fully characterized. Only then can long-range correlations be robustly tested and realized.

Although compositional databases have developed in tandem with the advances in cryptotephra discoveries, the archived data remain incomplete as submission depends on the initiative of the data ‘owner’. There is much to be gained from a community-wide effort to integrate tephra data on a much larger scale than previously felt necessary (Kuehn *et al.*, 2013). With Alaskan-sourced tephras identified in northern Europe, Pacific Arc tephras found in Greenland and Indonesian tephras found in East Africa, cryptotephra analysts have to widen their search in pursuit of a correlation. We can expect to see many developments in this area in the future. Additionally, as isochron maps and site networks are extended it is anticipated that the dispersal patterns will be of interest to the volcanology and atmospheric modelling communities (e.g. Stevenson *et al.*, 2013, 2012, 2015). As some of the far-travelled tephras are believed to originate from moderate-size eruptions (Davies *et al.*, 2010a; Jensen *et al.*, 2014), a challenge is now set to include cryptotephra occurrences within estimates of eruptive volume estimates and dispersal and transport models. It will be particularly interesting to explore whether such work will provide a revised eruptive volume or merely indicate the overriding influence of weather conditions at the time of eruption.

Finally, our future challenge is to fully exploit these cryptotephra deposits to undertake meaningful and insightful comparisons of the climatic and environmental records that they constrain. A full picture of past changes is only possible if high-resolution proxy records, anchored and dated by tephras and other dating techniques, can be integrated. Bronk Ramsey *et al.* (2014) are leading the way in this area and outline a database that is capable of integrating a range of quantified proxy datasets taking account of different timescales and their associated uncertainties as well as co-registered events, such as tephra or cryptotephra deposits. This web-based tool is a significant step towards optimizing and advancing the application of tephrochronology over the next 50 years and beyond.

## Endnotes

1 INTegration of Ice MArine and Terrestrial group. EU Cost Action 2010–2014 (ES0907). http://cost-es0907.geoenvi.org/

2 RESET: Response of Humans to Abrupt Environmental Transitions (UK NERC-funded Consortium) 2008–2013 http://c14.arch.ox.ac.uk/reset/embed.php?File=

3 TRACE: Tephra Constraints on Rapid Climatic Events (European Research Council funded starting grant) 2011–2016.

4 SMART: Synchronising Marine and Ice-core Records using Tephrochronology (UK NERC funded) 2009–2012.

## Abbreviations

INTAV: International focus group on tephrochronology and volcanism
JQS: Journal of Quaternary Science
XRF: X-ray fluorescence
